# The Most Common Clinical Features of Kawasaki Disease Patients in King Abdulaziz Medical City

**DOI:** 10.7759/cureus.15127

**Published:** 2021-05-19

**Authors:** Khalid Talal Alghamdi, Rahaf Abdulmalik Waggass, Syed Sameer Aga, Abdulaziz Ahmed Alrohaili, Ali Hassan Alaidroos, Mohammed Dakhilallah Alghamdi, Mohannd Khalid Algamdi, Ashwaq Talal Alghamdi

**Affiliations:** 1 College of Medicine, King Saud bin Abdulaziz University for Health Sciences, King Abdullah International Medical Research Centre, King Abdulaziz Medical City, National Guard Health Affairs, Jeddah, SAU; 2 Cardiology, King Saud bin Abdulaziz University for Health Sciences, Jeddah, SAU; 3 Basic Medical Sciences, College of Medicine, King Saud bin Abdulaziz University for Health Sciences, King Abdullah International Medical Research Centre, King Abdulaziz Medical City, National Guard Health Affairs, Jeddah, SAU; 4 Cardiology, King Abdulaziz University Faculty of Medicine, Jeddah, SAU

**Keywords:** kawasaki disease (kd), vascular inflammation, vasculitis, king abdulaziz medical city, saudi arabia

## Abstract

Background

Kawasaki disease (KD) is an acute idiopathic vasculitis affecting the small and medium-sized arteries especially coronary artery (CA). it occurs in childhood mostly below five years of age.

Objective

This study aims to identify the most common clinical features among KD patients in King Abdulaziz Medical City in Jeddah, Saudi Arabia from 1/1/1982 to 31/12/2018.

Methods

A case series study was conducted in all KD patients that were admitted to the King Abdulaziz Medical City from 1/1/1982 to 31/12/2018 except those who were diagnosed in other hospitals or whose diagnosis has later changed. The identification of patient was done by using the International Classification of Diseases (ICD) coding for KD (ICD9 446.1). Our data consisted of the patient’s file number, age at presentation, gender, whether the patients received IVIG treatment or not, number of days of fever before starting IVIG treatment, response to IVIG treatment, season in which the symptoms started and clinical features based on body’s system.

Result

The study included 18 patients, 11 males showed that (55.6%) of patients met the criteria of typical KD and most of them were less than five years old. In addition, most patients were reported to have polymorphous rash, cough, irritability, vomiting, and a murmur. All patients who received intravenous immunoglobulin (IVIG) treatment which demonstrated an improvement even though those who started the treatment after 10 days of fever.

Conclusion

In typical KD patients, the distribution of the clinical features was almost identical. However, there were some variations in them among atypical KD patients. Moreover, Evan though KD in our region is not common as in Japan, the incidence of giant aneurism was higher. In addition to that, this study and other study conducted in Saudi Arabia found that screened patients reported tachycardia more than patients in Japan.

Recommendation

As KD is still idiopathic, we recommend more details to be collected from the patients, especially consanguinity as it is common in Saudi Arabia

## Introduction

Kawasaki disease (KD) is an acute idiopathic vasculitis occurs mainly in children under five years of age, affecting small- and medium-sized arteries, especially coronary arteries. KD was initially reported by Tomisakau Kawasaki in 1967 in Japan; and thereafter, KD was recognized all over the world [1,2]. KD is characterized by sentinel features of fever that lasts for at least five days, cervical lymphadenopathy, bilateral conjunctivitis, oral mucosal changes, polymorphous rash, and swelling or redness of extremities [3,4]. Accordingly, KD is classified into two groups: (A) complete (typical) KD, which is defined by the presence of at least four of the five symptoms in addition to the fever [1]; and (B) incomplete (atypical) KD, defined by appearance of at least two of the five symptoms in addition to the fever [1]. Coronary arterial lesions are the most important complication of KD that may eventually lead to myocardial infarction [5]. KD is associated with other conditions such as mild hepatitis, aseptic meningitis, and pericardial effusion [3]. Intravenous immunoglobulin (IVIG) and aspirin are the treatment regimen used in KD to decrease the percentage of developing coronary arterial lesions [1].

Numerous studies around the world have reported similar clinicopathological characteristics of KD [6,7]. KD has been found to be more prevalent in Asian countries, especially in Japan, with an annual incidence of 308 per 100,000 children of less than five years of age, while in Europe incidence ranges between 4.9 and 15.2 per 100,000 children of less than five years of age [8]. Locally in Saudi Arabia, children of five years old incidence were reported as 7.4 per 100,000 [1].

Since there is limited data available for KD in Saudi Arabia, we carried out this case series to identify the clinical characteristics of patients affected by KD in King Abdulaziz Medical City in Jeddah, Saudi Arabia and to compare them with other local and international published data.

## Materials and methods

This case series study was undertaken in the Pediatrics Department of King Abdulaziz Medical City, National Guard Health Affairs in Jeddah, Saudi Arabia. The study was approved by the Institutional Review Board (IRB) of the hospital. The identification of the KD was confirmed by using the International Classification of Diseases (ICD) coding for KD (ICD9 446.1) retrospectively on all the children that were admitted to the Hospital from 1/1/1982 to 31/12/2018. Individual patient data were reviewed to confirm the diagnosis of KD according to the American Academy of Pediatrics and the American Heart Association (AHA) guidelines established in 2004. The patient’s demographic, clinical, laboratory and echocardiographic data were recorded.

We collected the data by using patients’ old system files. Our data consisted of the patient’s file number, age at presentation, gender, whether the patients received IVIG treatment or not, number of days of fever before starting IVIG treatment, response to IVIG treatment, season in which the symptoms started and clinical features based on body's system. All patients less than 15 years old who were diagnosed with KD were included in this study, except those patients who were diagnosed in other hospitals or whose diagnosis has later changed. Coding sheets were used to protect the identities of patients. Each patient was given a code linked to his/her medical record number (MRN) to preserve the patient’s information. 

Statistical analysis

Excel was used for data entry, and Statistical Package for the Social Sciences (SPSS) version 23 (IBM Corp., Armonk, NY) was used for calculating the mean and standard deviation for quantitative data, frequency and percentage for qualitative data.

## Results

The study included 18 patients, 11 of them were males. The male-to-female ratio was 1.6:1. Ten patients (55.5%) met the criteria of typical KD while the rest were diagnosed as atypical KD. Moreover, 14 patients (77.7%) found to be five years old or less while the rest were above the age of five years. The mean age was 52 months for males and 43 months for females.

All patients who received IVIG treatment showed improvement regardless of the number of days of fever before starting the treatment. However, there were two male patients who did not receive IVIG treatment (Table 1).

**Table 1:** IVIG treatment. IVIG: intravenous immunoglobulin.

Gender (No.)	Male (11)			Female (7)		
Management	Received IVIG treatment		Did not receive IVIG treatment	Received IVIG treatment		Did not receive IVIG treatment
	9		2	7		0
Number of days of fever before starting IVIG treatment	Less than 10 days	More than 10 days	-	Less than 10 days	More than 10 days	-
	6	3	-	4	3	-

As demonstrated in Table 2, the most common respiratory symptom related to KD was cough, and there were two patients reported to have upper respiratory tract infection. Bronchial asthma was found in two patients and nine patients were reported to have cardiovascular symptoms. The most common sign was a murmur and a giant aneurysm was reported in one patient. Cardiovascular symptoms were absent in nine patients which is 50% of the total number of patients. Regarding the musculoskeletal system, two patients were suffering from arthralgia and one of the patients reported other symptoms like arthritis, skeletal dysplasia, and kyphoscoliosis. Moreover, irritability was the commonest symptom of the nervous system with one of the patients was reported with torticollis. Eleven patients were having gastrointestinal symptoms of which the most common was vomiting found in 6 patients. Other gastrointestinal symptoms including abdominal pain and diarrhea, both in four patients. For the seasonal distribution of incidence, the number of cases in summer was 6 (33.3%), which was the highest, followed by spring and autumn with 5 (27.7%) patients for each, lastly the winter with 2 (11.1%) patients. There were no deaths reported.

**Table 2:** Clinical features of the patients. *These are the most common symptoms.

Gender (No.)	Male (11)		Female (7)		
Clinical features	Present	Absent	Present	Absent	
Classical criteria					
Acute lymphadenopathy	7 (64%)	4 (36%)	4 (57%)		3 (43%)
Conjunctival injection	7 (64%)	4 (36%)	3 (43%)		4 (57%)
Polymorphous rash	8 (73%)	3 (27%)	7 (100%)		0 (0%)
Lips and oral mucosal changes	6 (55%)	5 (45%)	6 (86%)		1 (14%)
Change in extremities (Swelling or erythema)	8 (73%)	3 (27%)	6 (86%)		1 (14%)
Other features					
Respiratory symptoms (Cough, rhinorrhea, throat congestion)*	5 (45%)	6 (55%)	3 (43%)		4 (57%)
Cardiovascular symptoms (Murmur, tachycardia)*	5 (45%)	6 (55%)	4 (57%)		3 (43%)
Musculoskeletal symptoms (Arthritis, arthralgia)*	3 (27%)	8 (73%)	0 (0%)		7 (100%)
Nervous system symptoms (Irritability, lethargy)*	2 (18%)	9 (82%)	3 (43%)		4 (57%)
Gastrointestinal symptoms (Vomiting, decreased oral intake)*	8 (73%)	3 (27%)	3 (43%)		4 (57%)
Dermatological symptoms (Rash, skin peeling)*	10 (91%)	1 (9%)	6 (86%)		1 (14%)
Hematological symptoms (Leukocytosis, thrombocytosis)*	3 (27%)	8 (73%)	1 (%17)		6 (%83)

## Discussion

King Abdulaziz Medical City is one of the largest hospitals in Jeddah. Among the 18 patients, 10 (55.6%) of them were typical for KD and 8 (44.4%) presenting with atypical KDs. The number of Our patients was small but close to other studies in the region. [1,7] The distribution of symptoms was almost identical in typical KD patients. However, in atypical KD patients, the most common symptom was polymorphous rash while it was not reported as the commonest feature of KD in other researches [5,7]. Moreover, the least reported symptom was conjunctival injection. However, studies in east Asia show that the conjunctival injection was the most common reported classical symptom [9,10]. In contrast to some of the European and most Asian studies where the maximum incidence occurred in winter, in our patients the highest incidence was in summer and the least in winter [11,12].

After dermatological changes which are included in the classical features of KD, gastrointestinal symptoms were the most reported finding in this study and also in another local study [7]. Vomiting was the highest noted symptom in our research, much like that found in Japan study as well as locally in Saudi Arabia [6,7,8]. Decreased oral intake was reported in 27.7% (five patients) compared to 37.5% in a local research study [7]. In addition, some research studies reported organomegaly. Only one of our patients was reported with splenomegaly [3,7]. As opposed to a research study that has been done in Spain, irritability was the least common symptom while being the highest in the research findings of Spain [8]. In our research, Even though we have only 18 patients one of them reported with giant aneurysms, which represent (5.6%). While in Japan, only (0.24%) had giant aneurysms [13]. Moreover, our patient was female in contrast to other research that showed that males have higher occurrence of giant aneurysms [8]. In our study, the incidence of tachycardia was 11.11%, which was 49% in another local study [1]. On the other hand, the incidence in japan was 0.07%. This suggests that Saudi Arabia has a higher incidence of tachycardia in this disease.

Except for two patients, all other patients received IVIG treatment, and regardless of the duration of fever before receiving IVIG treatment, all cases demonstrated an improvement; however, it is not suggested to delay the treatment with IVIG because most research studies have shown a better response rate if started prior to 10 days of fever [7,8,14]. No deaths were reported in our study. Most other studies even with large number of cases have also reported zero or a low death rate [8,9,13].

## Conclusions

In conclusion, since KD is rare in our region, physicians should have a high index of suspicion for this diagnosis. The etiology and pathogenesis of KD are not fully established, so we recommend better reporting of symptoms and clinical findings in patients with KD, particularly because it is common. Being a case series study in one center was a limitation in our study and it is recommended to perform a prospective multi-center study in the future to obtain added information pertaining to our nation.
